# DrugDomain 2.0: comprehensive database of protein domains-ligands/drugs interactions across the whole Protein Data Bank

**DOI:** 10.1101/2025.07.03.663025

**Published:** 2025-07-07

**Authors:** Kirill E. Medvedev, R. Dustin Schaeffer, Nick V. Grishin

**Affiliations:** 1 Department of Biophysics, University of Texas Southwestern Medical Center, Dallas, TX 75390, USA; 2 Present address: Department of Computer Science, University of Central Florida, Orlando, FL, 32816, USA; 3 Department of Biochemistry, University of Texas Southwestern Medical Center, Dallas, TX 75390, USA

**Keywords:** Small molecules, Drug discovery, Protein-drug interaction, Protein domains, Drugs, Database

## Abstract

Proteins carry out essential cellular functions – signaling, metabolism, transport – through the specific interaction of small molecules and drugs within their three-dimensional structural domains. Protein domains are conserved folding units that, when combined, drive evolutionary progress. The Evolutionary Classification Of protein Domains (ECOD) places domains into a hierarchy explicitly built around distant evolutionary relationships, enabling the detection of remote homologs across the proteomes. Yet no single resource has systematically mapped domain-ligand interactions at the structural level. To fill this gap, we introduce DrugDomain v2.0, the first comprehensive database linking evolutionary domain classifications (ECOD) to ligand binding events across the entire Protein Data Bank. We also leverage AI-driven predictions from AlphaFold to extend domain-ligand annotations to human drug targets lacking experimental structures. DrugDomain v2.0 catalogs interactions with over 37,000 PDB ligands and 7,560 DrugBank molecules, integrates 6,000+ small-molecule–associated post-translational modifications, and provides context for 14,000+ PTM-modified human protein models featuring docked ligands. The database encompasses 43,023 unique UniProt accessions and 174,545 PDB structures. The DrugDomain data is available online: https://drugdomain.cs.ucf.edu/ and https://github.com/kirmedvedev/DrugDomain.

## 1. Introduction

Studying how small molecules and drugs interact with protein structural domains lies at the heart of understanding both molecular function and guiding drug discovery. Through the binding of endogenous cofactors, metabolites, or exogenous drugs within their structural three-dimensional domains, proteins participate in a variety of vital cellular processes, including signaling, metabolism, and transport. Protein domains are conserved structural, functional, and evolutionary units that serve as the essential building blocks for protein diversity and adaptation [1]. The different ways in which protein domains can be combined provide a powerful mechanism for evolving new protein functions and shaping cellular processes [2]. Identifying and categorizing protein domains based on their evolutionary relationships can enhance our understanding of protein function. This is achieved by examining the established functions of their homologs. Until recently, major structure-based classifications of protein domains were primarily centered on categorizing experimentally determined protein structures, e.g., SCOP [3] and CATH [4]. Our team has developed and maintains the Evolutionary Classification of Protein Domains database (ECOD), whose key feature is its emphasis on distant homology, which culminates in a comprehensive database of evolutionary relationships among categorized domains topologies [5, 6]. Mapping the protein-ligand interactions at the domain level can reveal the mechanistic basis of protein function and inform structure-based drug discovery.

Artificial intelligence provides powerful tools for scientific research across diverse fields, and structural computational biology is no exception. AlphaFold (AF) has revolutionized structural biology by demonstrating atomic-level precision in protein structure prediction and becoming an indispensable tool in the field [7]. Leveraging AF models, ECOD stands out as one of the first databases to provide comprehensive domain classifications for both the entire human proteome [8] and the complete proteomes of 48 additional model organisms [6]. Recently The Encyclopedia of Domains (TED) [9] was launched - a comprehensive resource for the identification and classification of protein domains within the AlphaFold Database [10]. This advancement by AlphaFold has significantly broadened the scope of computational structural biology, enabling diverse applications such as drug discovery, drug target prediction, and the analysis of protein-protein and protein-ligand interactions [11, 12]. The new release of AlphaFold3 has further improved the accuracy of protein structure and protein-ligand interaction predictions [7].

As of today, no available resource reports interactions between protein structural domains (based on evolutionary classification) and ligands. With the latest advances in AI-based methods for protein structure and protein-ligand interaction predictions, we are witnessing a paradigm shift where computational approaches achieve performance levels nearly comparable to experimental methods. Here we present DrugDomain v2.0 (https://drugdomain.cs.ucf.edu/), a comprehensive database detailing the interactions of structural protein domains with a wide array of small organic (including drugs) and inorganic compounds, covering the full breadth of the Protein Data Bank. The database also provides domain-drug interactions for AlphaFold models of human drug targets without solved experimental structures [13]. It also features over 6,000 small molecule binding-associated PTMs and more than 14,000 PTM-modified human protein models with docked ligands [14]. In total, the database now encompasses 43,023 unique UniProt accessions, 174,545 PDB structures, 37,367 PDB ligands, and 7,561 DrugBank molecules. We believe this resource can serve as a foundation for a range of forward looking studies – including drug repurposing, the development of improved docking protocols, and the analysis of post translational modifications in protein-ligand interactions.

## 2. Materials and Methods

### 2.1. Data collection and analysis

The comprehensive list of ligands and small molecule components found in Protein Data Bank [15] was retrieved from Chemical Component Dictionary [16]. All PDB entries containing these ligands and small molecules’ InChI Key and SMILES formulas were obtained using rcsb-api [17]. Using InChI Keys and SMILES, we retrieved accession numbers for each small molecule from the following databases, where available: DrugBank [18], PubChem [19], ChEMBL [20]. In DrugDomain database, we use PDB ligand ID as a primary identified for the small molecule (for example NAD, 2I4, etc.). Alternatively, we use DrugBank accession for cases when the PDB ligand ID is unknown. Additionally, drug action data was retrieved from DrugBank and affinity data – from BindingDB [21]. Chemical classification of small molecule components was obtained from ClassyFire database [22] and include the four top levels of the classification: kingdom, superclass, class and subclass. 2D diagrams of ligand-protein interactions (LigPlots) were generated using LigPlot+ [23].

For each ligand-protein (PDB structure) pair, residues located within 5 Å of the atoms of the small molecule were identified using BioPython [24]. Interacting residues were mapped to structural domains from ECOD database v292 (08302024) [5] and reported in DrugDomain database. For ligand–protein pairs lacking experimentally determined structures, we used AlphaFold models and the AlphaFill algorithm [25] to transplant missing ligands from PDB structures into these models based on sequence and structural similarity. The methodology and implementation of this approach into DrugDomain database was described previously [13]. To calculate ligand-interacting statistics based on the number of domains, we counted the UniProt-accessioned proteins that included a specific number of ECOD domains interacting with the ligand. When multiple PDB structures corresponded to a single UniProt accession, the largest number of domains identified in any one of these structures was taken into account.

## 3. Results and Discussion

### 3.1. DrugDomain v2.0 statistics and features

DrugDomain v2.0 includes following major types of data related to interactions between protein domains and small molecule components. First, the new version of DrugDomain database reports domain-ligand interactions for all PDB entries containing ligand entities, including both organic small molecules and inorganic components. Thus, we expanded the scope of the database to encompass not only protein-drug interaction but also interactions between protein domains and all ligand entities that are present in PDB. Second, the v2.0 reports domain-drug interactions for AlphaFold models of human drug target proteins lacking experimentally determined structures [13]. Third, it includes over 6,000 small molecule binding-associated PTMs identified in human proteome and over 14,000 PTM-modified human proteins with docked ligands generated using recent AI-based approaches (AlphaFold3 [7], RoseTTAFold All-Atom [26], Chai-1 [27]) [14]. To help user navigate between different types of data we created detailed tutorial (https://github.com/kirmedvedev/DrugDomain/wiki/DrugDomain-database-Tutorial).

**Figure 1.**
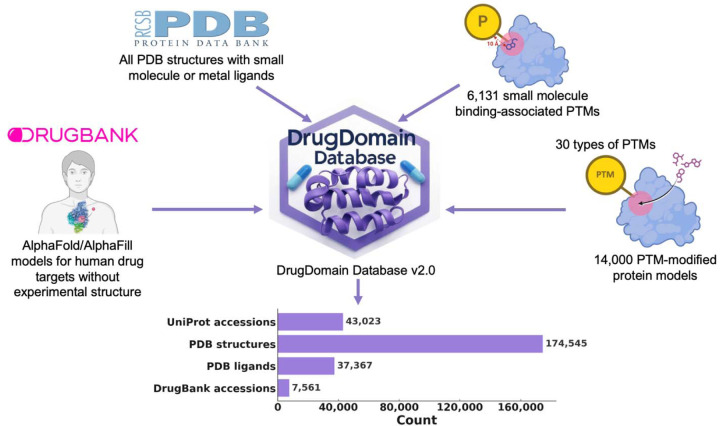
DrugDomain database v2.0 data types and statistics.

DrugDomain database v2.0, includes 43,023 unique UniProt accessions [28], 174,545 PDB structures (over 70% of all experimental protein structures), 37,367 ligands from PDB, 7,561 DrugBank molecules (over 50% of all small molecule drugs in DrugBank) (Fig. 1).

DrugDomain includes two types of hierarchy: protein and molecule-centric. The complete lists of proteins and small molecules can be accessed through the top menu. There are two types of molecule lists – by DrugBank accession and by PDB ligand id. The protein or molecule can be searched using the search field on the main page. The search can be conducted using UniProt (e.g. Q03181), PDB ligand (e.g. ATP) or DrugBank accessions (e.g. DB00171). The search by UniProt accession returns a list of ligands known or predicted to interact with the query protein, along with key data for each ligand: PDB ID; DrugBank, PubChem, and ChEMBL accessions; molecule name; drug action; and affinity. The molecule search (by PDB ligand ID or DrugBank accession) returns a list of proteins known or predicted to bind the query molecule, along with key data for ligand and protein. Both search types return links to DrugDomain data pages, which provide key ligand information, including its chemical classification, and list PDB structures and/or AlphaFold models known or predicted to bind the ligand. The list of the structures includes PDB/AF accession, downloadable PyMOL [29] script, which shows ECOD domains and residues interacting with the ligands; a list of ECOD domains interacting with the molecule with links to the ECOD database, names of corresponding ECOD X-groups (possible homology level) and 2D diagrams of ligand–protein interactions (LigPlots). DrugDomain data webpage also includes link to list of drug binding-associated post-translational modifications (PTMs) where available [14]. This list includes information about each PTM and links to PyMOL sessions with models of modified proteins generated by AlphaFold3, RoseTTAFold All-Atom or Chai-1. PyMOL sessions include mapped ECOD domains shown in various colors and modified residue and ligand.

**Figure 2.**
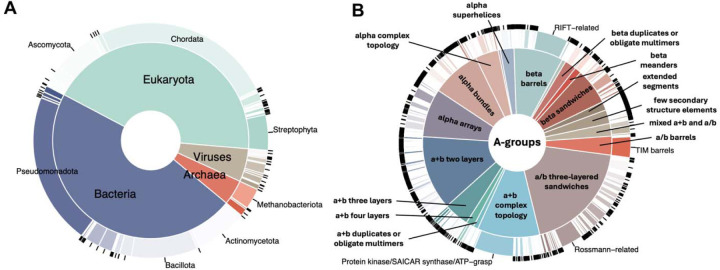
DrugDomain v2.0 statistics. **(A)** Taxonomic distribution of proteins reported in the DrugDomain database, by UniProt population. The inside pie shows the distribution of superkingdoms, and the outside donut shows the distribution phyla. **(B)** Distribution of ECOD domains from experimentally determined PDB structures, interacting with ligand, stratified by architecture (inside pie) and homologous group (outside donut).

The taxonomic distribution of proteins reported in the DrugDomain database v2.0 revealed prevalence of eukaryotic and bacterial proteins (Fig. 2A). *Pseudomonadota* or proteobacteria are one of the most abundant phyla of Gram-negative bacteria, which are naturally found as pathogenic and free-living genera [30]. Thus, proteins from these bacteria are important targets for antibacterial therapy against human pathogens, and PDB entries of these proteins bound to various antibiotics comprise a significant fraction of the Protein Data Bank. Bacteria belonging to the phylum *Bacillota* can make up 11–95% of the human gut microbiome [31] and play key roles in energy extraction. They have also been associated with the development of diabetes and obesity [32], making them potential therapeutic targets. Finally, the third-largest phylum in terms of the number of PDB structures with ligands is *Actinomycetota* (or Actinobacteria). These bacteria are major contributors to biological buffering of soils and the source of many antibiotics [33]. Similarly, there are three largest eukaryotic phyla: Chordata includes human and various model organisms such as mice and rats; Ascomycota is a largest phylum of fungi, which are the source of antibiotics like penicillin, and particular species are used to produce immunosuppressants and other medicinal compounds [34]; *Streptophyta* phylum includes green algae and the land plants.

The distribution of ECOD domains from experimental structures interacting with ligands is shown in Figure 2B. The top three largest ECOD A-groups include α/β three-layered sandwiches, α+β two layers and α+β complex topology. The α/β three-layered sandwich architecture is represented mainly by Rossmann-like proteins. In our earlier work, we showed that these proteins perform diverse functions and interact with most superclasses of organic molecules [35, 36]. Most small molecules that interact with domains of the α+β complex topology target protein kinases, which are among the most druggable proteins in the human proteome; therefore, their structures are abundant in the Protein Data Bank [37, 38]. The α+β two-layers architecture includes heat shock proteins (HSP), which play a critical role as molecular chaperones and are important targets for anticancer chemotherapy [39].

**Figure 3.**
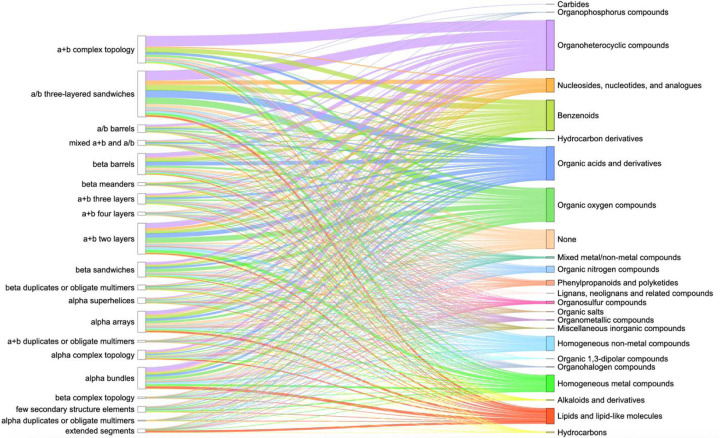
ECOD A-groups (left column) of experimental PDB structures and superclasses of organic molecules according to ClassyFire classification (right column). Each superclass and the lines pointed toward it are denoted by separate color. The thickness of the lines shows the number of PDB ligands interacting with domains from ECOD A-groups.

The distribution of domains of experimental PDB structures and superclasses of organic compounds they interact with revealed the top three most common superclasses of ClassyFire classification [22] in Protein Data Bank: Organoheterocyclic compounds, Organic oxygen compounds, Organic acids and derivatives (Fig. 3). The largest fraction of domains interacting with compounds from the majority of superclasses belongs to α/β three-layered sandwiches, α+β two layers and α+β complex topology ECOD architecture types, which were discussed above. The superclass Organoheterocyclic compounds includes atorvastatin, a lipid-lowering drug that reduces the risk of myocardial infarction, stroke, and other cardiovascular diseases [40]. Erythromycin is a broad-spectrum antibiotic in the Organic oxygen compounds superclass and is widely used to treat infections caused by both Gram-positive and Gram-negative bacteria [41]. Finally, Arbaclofen – a member of the Organic acids and derivatives superclass - is a drug that is used in the treatment of autism [42].

### 3.2. Number of domains mediating ligand interactions in Protein Data Bank

**Figure 4.**
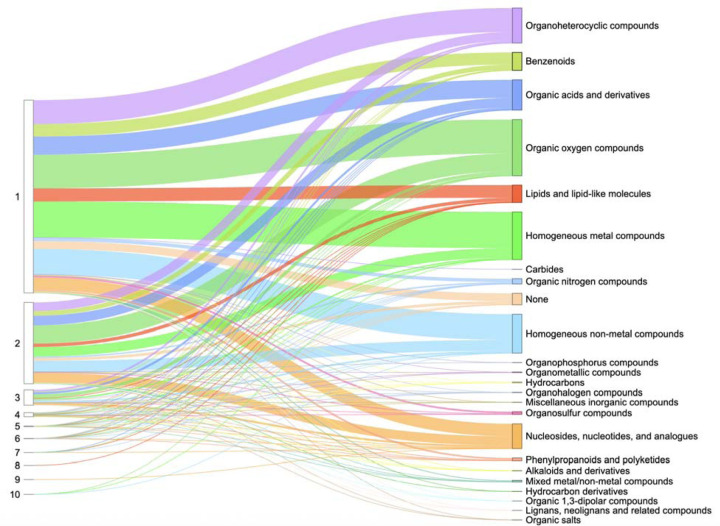
Ligand-interacting statistics by number of domains per UniProt accession in Protein Data Bank. The left column shows the number of ligand-interacting domains, the right column shows the superclasses of organic molecules according to ClassyFire classification. The thickness of the lines shows the number of UniProt accessions.

Protein domains are conserved structural units that serve as the fundamental evolutionary and architectural building blocks of proteins. Understanding how ligands bind – specifically, which domains are involved and how many mediate the interaction – is crucial for uncovering protein function and guiding drug discovery. Overall ligand-interacting statistics were calculated for each protein, based on the number of interacting ECOD domains associated with its UniProt accession (Fig. 4). Our results revealed that the majority of proteins with assigned ECOD domains bound ligands using one or two domains. Our observation is consistent with previous research, which indicates that most drug targets bind via a limited set of prevalent domains [43]. Moreover, it is noteworthy that, under the ECOD classification, protein kinases – the most druggable targets in the human proteome – are characterized by a single structural domain [38]. This contributes to their significant representation among proteins with one ligand-interacting domain. In contrast, other structural classifications divide these proteins into two domains [4]. It is important to note, however, that experimentally determined PDB structures may not always accurately reflect ligand coordination, as only a part of the protein is often included in the experimental structure.

**Figure 5.**
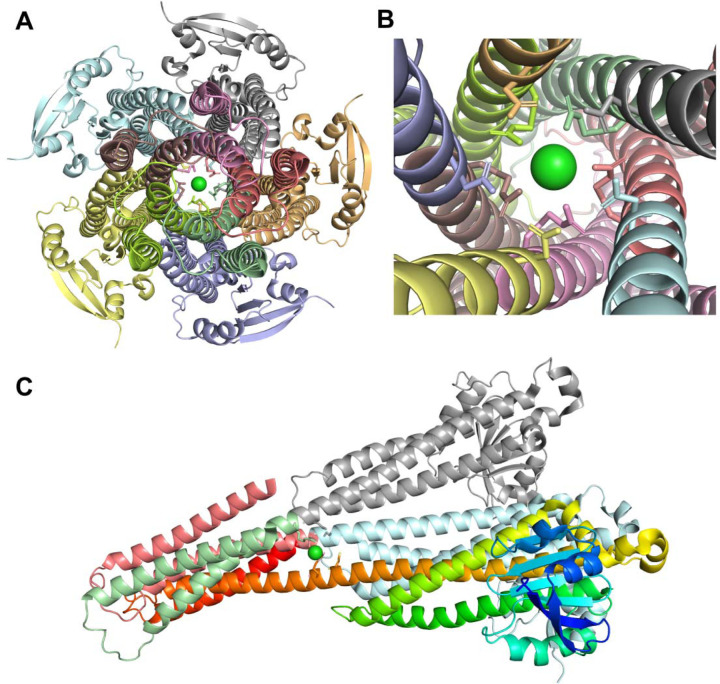
Structure of the human mitochondrial Mrs2 channel (PDB: 8IP5). **(A)** Channel view of Mrs2 with protein colored by ECOD domains, Mg ion is shown in green, and sticks show interacting residues. **(B)** Close-up channel view of Mrs2. **(C)** Side view of Mrs2 showing three out of five monomers. Two chains are colored by ECOD domains, one – by rainbow from blue (N-terminal part) to red (C-terminal part).

**Figure 6.**
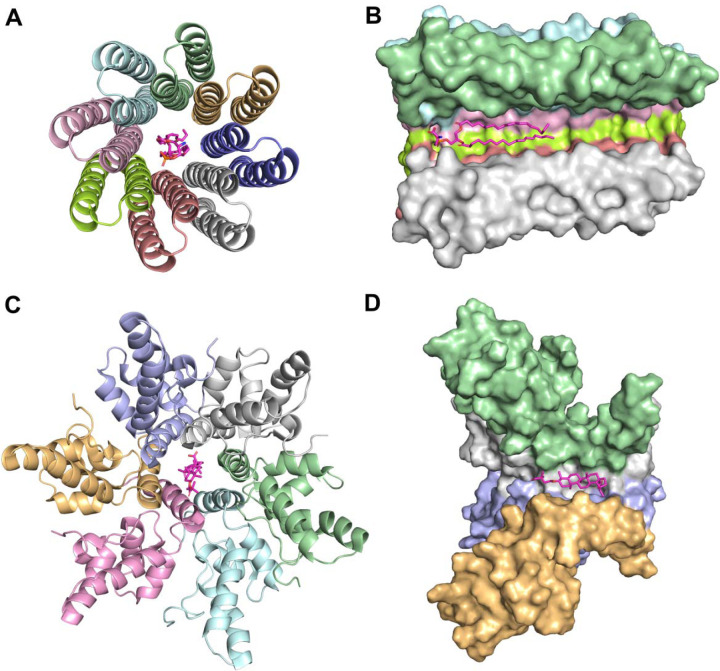
Examples of small molecules coordinated by pore structures. **(A)** Pore view of the c-ring of mammalian F-type ATP synthase (PDB: 6TT7). Phosphatidyl serine is colored in magenta. Protein structure is colored by ECOD domains. **(B)** Side view of the c-ring. Two chains were removed. **(C)** Pore view of the HIV-1 Gag polyprotein (PDB: 7R7P) with inhibitor Bevirimat (DrugBank: DB06581) colored in magenta. Protein structure is colored by ECOD domains **(D)** Side view of the HIV-1 Gag polyprotein. Two chains were removed.

Our analysis of ligand-interacting statistics indicated that proteins deposited in the Protein Data Bank contain a range of one to ten ECOD domains involved in ligand interaction (Fig. 4). Such a large number of interacting domains (ten) can bind a single ligand when the protein forms a channel or pore structure. For example, human mitochondrial RNA splicing 2 (Mrs2) channel (Fig. 5A–C) enables Mg^2^ permeation across the inner mitochondrial membrane and is crucial for mitochondrial metabolic function [44], illustrating how a channel structure can accommodate interactions with multiple domains. Dysregulated Mg^2^ levels in humans are implicated in various diseases [45], as mitochondria are the primary site of ATP production in eukaryotic cells – a process critically dependent on Mg^2^ as a cofactor. The cation also commonly forms complexes with cellular nucleotides [46]. Mrs2 exists as homopentamers, with each monomer featuring two C-terminal transmembrane helices [46]. Structurally, each monomer contains two ECOD domains: an N-terminal “CorA soluble domain-like” domain and a C-terminal transmembrane domain (Fig. 5C). Mg^2^ is coordinated near the borders of two domains of each monomer and interacts with each domain of homopentamer (Fig. 5B). However, not only metal ions can be coordinated by the channel or pore structure. The c-ring of mammalian F-type ATP synthase is an annular rotor composed of multiple identical c-subunits (eight in mammals) embedded in the Fo membrane domain (Fig. 6A–B) [47]. Protons enter via the interface between the a-subunit stator and the c-ring, bind to specific residues on a c-subunit, and are then carried around as the ring turns, before being released on the opposite side of the membrane. This proton driven rotation of the c-ring transmits torque through the central stalk (γ-subunit) to the F1 catalytic domain, driving ATP synthesis [47]. It was discovered that in mammalian F-type ATP synthase, subunit e caps the c-ring via a lipid “plug”. Figures 6A and 6B depict phosphatidylserine acting as the c-ring plug. The c-ring is a homooctamer, with each monomer featuring a single ECOD domain. Figures 6C and 6D depict Bevirimat, an inhibitor of HIV-1 maturation, bound to the HIV-1 structural Gag polyprotein. HIV-1 maturation occurs when the viral protease cleaves the Gag polyprotein precursor into its constituent domains [48]. HIV-1 maturation inhibitors exploit a protective mechanism whereby the Gag polyprotein’s critical cleavage site is located within a stable six-helix bundle (Fig. 6C). Bevirimat binds to this junction, stabilizing the bundle and preventing it from unfolding, which is necessary for the viral protease to access and cleave the polyprotein [49]. The six-helix bundle unit forms hexamer with each monomer featuring a single ECOD domain (Fig. 6C–D).

## Conclusions

The DrugDomain database version 2.0 represents a comprehensive resource depicting interactions between structural protein domains and small organic (including drugs) and inorganic molecules across the entire Protein Data Bank. It also reports domain-drug interactions for AlphaFold models of human drug targets lacking experimental structures. Additionally, it features over 6,000 small-molecule binding-associated PTMs and more than 14,000 PTM-modified human protein models with docked ligands, generated by state-of-the-art AI-based approaches. DrugDomain database v2.0, includes 43,023 unique UniProt accessions, 174,545 PDB structures, 37,367 ligands from PDB, 7,561 DrugBank molecules. Within experimental PDB structures, the distribution of ECOD domains interacting with ligands was analyzed. This analysis revealed that the top three ECOD A-groups, ranked by the number of ligand-interacting domains, are predominantly α/β three-layered sandwiches (Rossmann fold), α+β two layers (heat shock proteins) and α+β complex topology (kinases). The distribution of domains in experimental PDB structures and their interacting compound superclasses identified the top three categories as Organoheterocyclic compounds, Organic oxygen compounds, and Organic acids and derivatives. Our analysis showed that proteins in the Protein Data Bank exhibit a range of one to ten ECOD domains involved in ligand interaction. All data and protein models are available for view and download in the DrugDomain database (https://drugdomain.cs.ucf.edu/) and GitHub (https://github.com/kirmedvedev/DrugDomain).
